# Cutting-Based Manufacturing and Surface Wettability of Microtextures on Pure Titanium

**DOI:** 10.3390/ma17153861

**Published:** 2024-08-04

**Authors:** Haoyu Li, Yuanjin Cong, Shuai Zhou, Junjie Zhang

**Affiliations:** Center for Precision Engineering, Harbin Institute of Technology, Harbin 150001, China; haoyu.li0219@gmail.com (H.L.);

**Keywords:** pure titanium, precision cutting, gird microtexture, wettability, finite element simulation

## Abstract

Pure titanium is a preferred material for medical applications due to its outstanding properties, and the fabrication of its surface microtexture proves to be an effective method for further improving its surface-related functional properties, albeit imposing high demands on the processing accuracy of surface microtexture. Currently, we investigate the fabrication of precise microtextures on pure titanium surfaces with different grid depths using precision-cutting methods, as well as assess its impact on surface wettability through a combination of experiments and finite element simulations. Specifically, a finite element model is established for pure titanium precision cutting, which can predict the surface formation behavior during the cutting process and further reveal its dependence on cutting parameters. Based on this, precision-cutting experiments were performed to explore the effect of cutting parameters on the morphology of microtextured pure titanium with which optimized cutting parameters for high-precision microtextures and uniform feature size were obtained. Subsequent surface wettability measurement experiments demonstrated from a macroscopic perspective that the increase in the grid depth of the microtexture increases the surface roughness, thereby enhancing the hydrophilicity. Corresponding fluid–solid coupling finite-element simulation is carried out to demonstrate from a microscopic perspective that the increase in the grid depth of the microtexture decreases the cohesive force inside the droplet, thereby enhancing the hydrophilicity.

## 1. Introduction

Pure titanium has emerged as a preferred material in biomedical application, aerospace, and other industries owing to its low density, high specific rigidity, outstanding corrosion resistance, and exceptional biocompatibility [1,2,3]. Especially in the field of biomedical applications, pure titanium is extensively utilized for producing medical devices, artificial joints, dental implants, etc. While surgical implants have high requirements for surface functionality, forming microscopic concave–convex microtextures on the surface is an effective method to improve surface functionalities such as wettability, biocompatibility, etc. The enhanced wettability increases the attachment of cell tissues, promotes their proliferation and differentiation, and also reduces the friction coefficient, thus improving the surface lubrication properties of the implant. Therefore, the fabrication of pure titanium surface microtextures can enhance wettability, which provides potential benefits for the application of surgical implants made of pure titanium in the biomedical field.

The accuracy of microtextures on surfaces is pivotal to the functioning of parts. It was found that wettability and biocompatibility are affected by multiple factors such as tool wear, material deformation and fracture, and surface roughness, which are in turn affected by the accuracy of the surface microtexture [4]. The optimization of the processing parameters for the fabrication of surface microtextures on aluminum alloys indicated that the processing accuracy of surface microtextures plays a vital role in improving optical and frictional properties [5]. Processing defects in the surface microtexture can significantly affect distribution density, morphology, geometry, etc., thus leading to serious implications for wettability and biocompatibility [6]. Research on the microtextures of stainless steel surfaces demonstrate that the accuracy of surface microstructures can bring significant improvements to the optical, tribological, and biocompatibility properties of materials [7,8]. Improving processing parameters to optimize surface roughness, relative densification, and the development of surface microtextures can also improve biocompatibility [9]. Therefore, it is imperative to employ appropriate processing techniques for preparing surface microtextures in order to ensure the material possesses a high-precision surface microstructure, thereby enhancing the performance and application versatility of the material.

Laser surface texturing (LST) technology is one of the regular methods of processing surface functional microtextures [10]. In terms of wettability, three different microtextures (linear, grid-like, and dot-like) were fabricated on titanium surfaces using nanosecond lasers, and it was found that the contact angle was significantly reduced and the hydrophilicity was enhanced after laser treatment [11]. Research on the effect of surface microtexture spacing on wettability indicated that as the surface microtexture spacing increased, the contact angle first increased and then decreased [12]. The surface contact angle on the laser-microtextured surface dropped from 94.8° ± 1.2° to 12.8° ± 1.7° in high-purity water [13]. The biomimetic hexagonal microstructure on the titanium surface increased the surface roughness but reduced the contact angle and enhanced the hydrophilicity [14]. Studies on the surface microtexture of TC4 formed by LST found that the microtexture had a significant effect on the wetting ability [15]. In terms of biocompatibility, a study using ultraviolet laser to process various surface microtextures on titanium alloy surfaces found that cells exhibited optimal adhesion and proliferation abilities on the surface with a surface roughness of 5 μm [16]. Research on the effects of microstructure aspect ratio and depth gradient on osteoblast adhesion properties found that osteoblasts within the microstructure exhibited optimal growth, proliferation, and adhesion at an aspect ratio of 2.5:1 and a maximum depth gradient of 15.7 μm [17]. The microtexture of grooves formed by LST on TC4 can effectively enhance biofunctionalization aspects such as contact guidance, cell adhesion, and cell growth [18]. Cells on microtextured TC4 surfaces exhibited a preference for distribution along the roughness peak areas of microtexture edges and cavities [19]. The surface microtexture on titanium alloy enhanced wettability and protein adsorption capacity [20]. However, LST also presented certain limitations. Firstly, LST involves material removal due to thermal effects, resulting in significant heat-affected zones around the microtextured areas, which potentially leads to defects formed by melt recasting. Additionally, the laser spot size typically ranges in the order of tens of microns, which can result in reduced accuracy and consistency in the microtexture.

Cutting processes fabricate microstructures by removing material through movements in relation to the ultra-small tool and workpiece, making it widely utilized due to its advantages of high-processing accuracy and efficiency, strong controllability, and wide machinability. A self-cleaning surface with a 3D-patterned microstructure was fabricated using ultra-precision processing technology, and a high-static water contact angle approaching 150° was successfully achieved [21]. The surface with nanoscale roughness was achieved by using offset-tool-servo flycutting, and an inverted pyramid microstructure array was machined through path planning [22]. A typical two-dimensional sinusoidal wave surface microstructure with a surface roughness of 7.49 nm was obtained by using a novel fast-tool servo technology that provides restoring force [23]. The sine-shaped and honeycomb-shaped microstructures with an average machining error of less than 3% were machined on the Ti surface using a slow-tool servo [24]. However, pure titanium is a challenging material to machine. During the cutting process, the low thermal conductivity and high thermal expansion coefficient of pure titanium resulted in increased temperature and thermal stress concentrations in the cutting area, leading to increased thermal damage and tool wear. Moreover, the high ductility of pure titanium increased its susceptibility to substantial plastic deformation, which was not conducive to effective material removal by cutting tools. Currently, there are limited reports on the preparation of surface microtextures through the cutting of pure titanium. Ultrasonic vibration cutting for pure titanium was used to fabricate surface microtextures with diamond-shaped and bamboo-shaped pits, and a prediction model for cutting force in ultrasonic vibration-assisted cutting (UVAC) was established [25]. The model established in the previous study of pure titanium UVAC is not universally applicable for the fabrication of the surface microtexture by pure titanium cutting. Therefore, it is essential to explore the cutting mechanisms specific to pure titanium materials and the dependence of surface microtextures on cutting parameters.

Finite element method (FEM) simulation is important to investigate the material cutting process [26]. The FEM simulation on the surface residual stress of TC4 under different cutting parameters indicated that the chip morphology directly affects the residual stress distribution on the chip surface [27]. The multiscale FEM simulation of cutting of Ti alloy demonstrated that the depth of the hardened layer decreases with the reduction of cutting speed and cutting depth [28]. The FEM simulation of ultrasonic vibration-assisted cutting of TC4 indicated that the residual stress compressive layer thickness increased with the increase of ultrasonic power and decreased with the increase of cutting speed [29]. The FEM simulation on the cutting process of TC4 based on the Euler-Lagrange coupled method demonstrated that the simulated ribbon chip was consistent with that obtained in the cutting experiment [30]. While the previous study predominantly concentrated on TC4, the FEM simulation applied to the pure titanium precision cutting is relatively scarce. An FEM simulation model for pure titanium cutting was developed to predict the evolution of microstructures in materials during the cutting process [31]. The FEM simulation of pure titanium cutting using a textured tool was carried out, and it was observed that the width of the microgrooves and the convexity significantly influenced the microstructure distribution in the material [32]. Although the above studies provide valuable perspectives on pure titanium cutting, the surface generation mechanism of pure titanium cutting is not yet understood. Therefore, it is needed to develop a pure-titanium cutting FEM simulation model with surface generation characteristics.

In the past decades, extensive studies based on the measuring of contact angles have been performed to investigate the wettability of microstructures. A contact angle measurement experiment was conducted on a titanium alloy with surface microtexture, and it was found that the contact angle of the surface microtexture decreased from the initial 40.25° to 9.88° [33]. The apparent contact angle distribution on surfaces patterned with micrometric pillars and grooves was measured, demonstrating that surface microtextures can enhance wettability [34]. Contact angle measurement experiments on microchannel copper plate surfaces found that the optimal droplet size was 9 μL, which can enhance the wettability across various microchannel surfaces [35]. The contact angle measurement experiments were conducted on titanium with surface microstructures in air and underwater, and it was found that the wetting behavior in underwater bubbles is closely related to the wettability in air [36]. However, the microscopic details and kinetic behaviors of the wetting process remain not fully understood. Thus, it is necessary to employ fluid–solid coupling FEM simulation to elucidate the wetting behavior of microtextures.

Therefore, in the current work, the fabrication of pure titanium surface microtextures was performed, along with a study of wettability. Firstly, an FEM simulation model of pure titanium cutting with surface generative characteristics was established, and the model accuracy was verified by combining it with the experiment. Based on this, the dependence of the surface generative mechanism on cutting parameters in the pure titanium cutting process was investigated by FEM simulation. Then, with this as the foundation, the process parameters were optimized and high precision grid microtextures with a pitch of 200 μm and different grid depths of 10 μm, 20 μm, and 30 μm were fabricated. Finally, the contact angle measurement experiment was employed to explore the effect of geometric structure parameters on the wettability of microtextured pure titanium surfaces, and the fluid–solid coupling FEM simulation was utilized to indicate the wettability behavior of microtextured pure titanium surfaces.

## 2. Materials and Methods

### 2.1. Experimental Setup of Pure Titanium Precise Cutting

**Figure 1:**
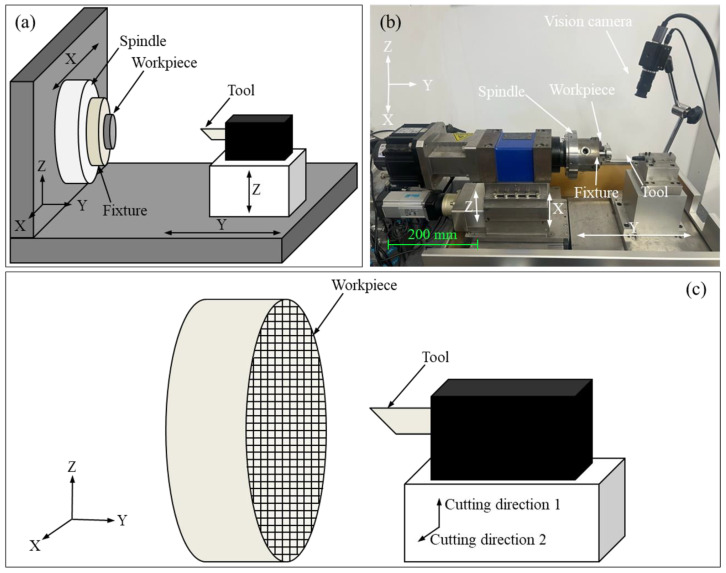
(**a**) Layout and (**b**) experimental configuration of four-axis machine tool; (**c**) Schematic diagram of fabrication process of grid microtexture.

In this study, the cylindrical workpiece fabricated from pure titanium exhibits a diameter of 30 mm and a thickness of 8 mm. Table 1 lists chemical constituents of pure titanium. Before cutting experiments, the workpiece of pure titanium is ground to make its surface flatness less than 5 μm. Subsequently, the ground workpiece undergoes ultrasonic cleaning in a liquid mixture of water and absolute ethanol for a duration of 10 min at room temperature to eliminate any pollutants. Figure 1 illustrates the four-axis machine tool utilized for the cutting experiments. The arrangement of the machine tool illustrated in Figure 1a is composed of a rotating spindle (C axis), the horizontal axis of X and Y, the vertical axis of Z, a workpiece fixture, a tool holder, and a cutting tool. The nose radius of the PCD tool and the carbide tool are 1 mm and 50 μm, respectively. Since the workpiece is positioned at the center of the spindle, slow-tool servo technology is employed to control the tool motion through the C-axis, *X*-axis, and *Z*-axis to achieve the fabrication of the grid microtexture on the pure titanium surface, as illustrated in Figure 1c. Furthermore, the share area is an important factor influencing the functionality of microtextured surfaces. The effect of microtexture geometry on the functional properties of microtextured TC4 surfaces such as wettability and friction properties is explored, and it was found that the grid microtexture with a pitch of 200 μm exhibits lower surface contact angles and coefficients of friction in all conditions than the micro-dimple texture of other sizes, which is attributed to its higher area share [37]. Thus, in this study, the grid microtexture with a pitch of 200 μm is considered. Three grid depths of 10 μm, 20 μm, and 30 μm are fabricated. After the cutting experiments, the machined workpiece with grid microtexture undergoes ultrasonic cleaning in a liquid mixture of water and absolute ethanol for a duration of 5 min at room temperature to eliminate any pollutants, and the grid microtexture profile is characterized by white light interferometer.

### 2.2. Experimental Setup of Surface Wettability Measurement

In the present study, the sessile drop measurement with distilled deionized water at room temperature is employed to evaluate the surface wettability of both non-microtextured and microtextured pure titanium workpieces, aiming to explore the effect of the grid microtexture geometric parameters on the pure titanium wetting. Prior to the sessile drop measurement, the workpieces are cleaned in a liquid mixture of water and absolute ethanol for 5 min at room temperature using ultrasonic treatment. During the sessile drop measurement, the 2 µL of distilled-deionized (DD) water is dropped on the dried workpiece surface using a micrometer syringe for a depositing time for 60 s, and the contact angle is recorded every 6 s. Three repeated sessile drop measurements at a consistent location are performed to ensure the precision of the experimental data.

### 2.3. FEM Modeling of Pure Titanium Cutting

**Figure 2:**
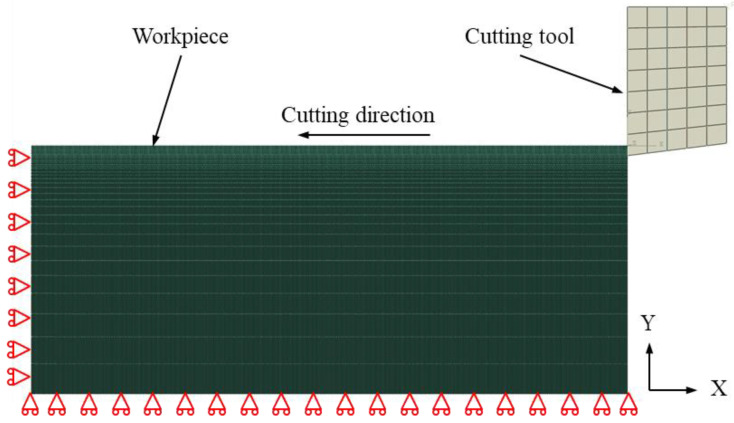
2D FEM model of pure titanium cutting.

Figure 2 shows the 2D FEM model of pure titanium orthogonal cutting, which consists of a workpiece and a cutting tool. The workpiece has dimensions of 300 μm in length and 125 μm in width, and its bottom and left sides are completely fixed. The modified Johnson–Cook (J–C) model of pure titanium is utilized and is represented by Equation (1) [38]:

(1) $$\sigma =\left[B\varepsilon^{n}\right]\left[1+Cln\left(\frac{\dot{\varepsilon }}{{\dot{\varepsilon }}_{0}}\right)\right]\left[\alpha e^{\beta \left(\frac{T_{melt}-T}{T_{melt}-T_{reference}}\right)}\right],$$

where σ is an equivalent stress, ε is a plastic strain, $\dot{\varepsilon }$ is a strain rate, and *T* is an operating temperature. The material parameters related to pure titanium are: *B* stands for hardening modulus, *n* for work hardening components, *C* for strain rate dependency coefficient, ${\dot{\varepsilon }}_{0}$ for reference strain rate, *α* and *β* for thermal softening coefficients, *T_reference_* for reference temperature, and *T_melt_* for melting temperature. The modified J–C model parameters of pure titanium are listed in Table 2.

The J–C damage model of pure titanium is adopted and is represented by Equation (2) [39]:

(2) $$\varepsilon_{f}=\left[D_{1}+D_{2}exp\left(D_{3}\sigma^{*}\right)\right]\left[1+D_{4}\ln\dot{\varepsilon_{p}^{*}}\right]\left[1+D_{5}T_{H}\right],$$

where $\varepsilon_{f}$ is an equivalent fracture strain, $\sigma^{*}$ is a stress triaxiality, and the material constants related to pure titanium are *D*_1_, *D*_2_, *D*_3_, *D*_4_, and *D*_5_. The parameters used in the J–C damage model are listed in Table 3.

The workpiece and cutting tool make contact using the surface-to-surface method, with a friction coefficient between them of 0.1. The workpiece is fully meshed by 4-node plane strain thermally coupled quadrilateral elements (CPE4RT), with the element size increasing monotonically from a minimum of 500 nm on the top surface to a maximum of 15 μm on the bottom surface. The tool is fully meshed by CPE4RT, with the element size of 10 μm. Since the hardness of the PCD tool and carbide tool are much higher than that of the workpiece of pure titanium, the cutting tool is set as a rigid body. In the cutting process, the pure titanium workpiece is fixed on the left side and bottom with full constraint, and the cutting tool operates in the negative X direction at different cutting depths and cutting speeds until the material is entirely removed.

### 2.4. FEM Modeling of Pure Titanium Surface Wettability

**Figure 3:**
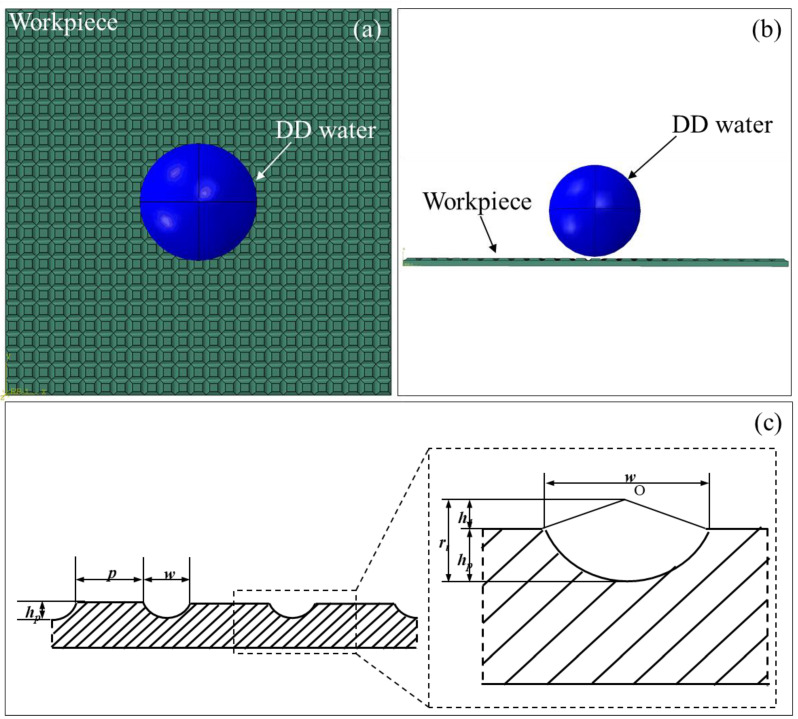
(**a**) Top view and (**b**) side view of 3D FEM model of wettability of microtextured pure titanium surface; (**c**) Representation of designed microtextured surface.

Figure 3a,b illustrate the top and side view of 3D FEM model of wettability testing on microtextured pure titanium surface, which is composed of microtextured pure titanium workpiece and DD water. To ensure the consistency with experiments, the simulated DD water has a volume of 2 µL and a diameter of 0.78 mm, which is meshed by 8-node linear brick elements (C3D8R) with a mesh size of 8 μm. The pure titanium workpiece is a square plate with 5 mm in side length and 1 mm in thickness. And the geometric parameters of the grid microtextures on the workpiece are consistent with the experimental parameters, with a pitch of 200 μm and different grid depths of 10 μm, 20 μm, and 30 μm, which are meshed by C3D8R with the minimum mesh size of 5 μm at the groove of the microtexture and the maximum mesh size of 50 μm at the workpiece edge. Furthermore, the total simulation time is 2 s to reveal the transient process of contact behavior between DD water and pure titanium workpiece in the initial stage of the wetting experiment.

The cutting tool geometry in terms of nose radius has a direct influence on the geometry of grid microtexture. The depth (*h_p_*), width (*w*), and pitch (*p*) of the fabricated microtexture are directly related to the tool nose radius (*r_t_*), cutting depth (*d*), and tool movement distance (*s*), as shown in Figure 3c. Therefore, the dimension of microtexture can be precisely controlled though the machine slides. However, the width (*w*) and pitch (*p*) of the microtexture are mainly limited due to tool geometry and can be calculated in terms of four tool parameters by Equations (3)–(6).

(3) $$h_{p}=d,$$

(4) $$h_{1}=r_{t}-h_{p},\;\mathrm{If}\;(r_{t}\;>\;h_{p}),$$

(5) $$w=2h_{1}\times \tan\left(\cos^{-1}\left(1-\frac{h_{p}}{r_{t}}\right)\right),$$

(6) p=s−w,

where *d* is grid depths of 10 μm, 20 μm, and 30 μm, and *s* is grid pitch of 200 μm. Be consistent with the wettability experiment, in the entire FEM simulation, the workpiece is fixed by holonomic constraint, and the entire model is affected by gravity in the *Z*-axis direction.

## 3. Results and Discussion

### 3.1. Cutting Behavior of Pure Titanium

**Figure 4:**
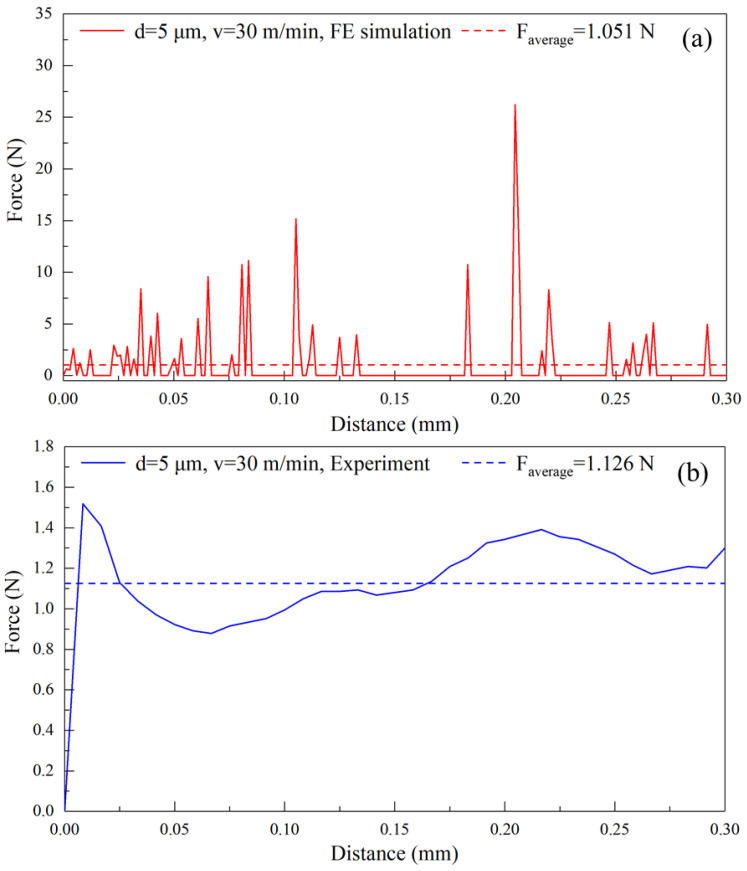
Variations of cutting force over cutting distance in (**a**) FEM simulation and (**b**) experiment of pure titanium cutting at a cutting depth of 5 μm and a cutting speed of 30 m/min.

The experiment and the FEM simulation are firstly carried out on pure titanium cutting at a cutting depth of 5 μm and a cutting speed of 30 m/min, aiming to explore the cutting performance of pure titanium. Figure 4 shows the variations of cutting force over the cutting distance in the FEM simulation and the experiment. In the FEM simulation, the simulated cutting force initially shows a rapid increase as the tool penetrates into the workpiece surface, followed by significant fluctuation around the average value. However, the fluctuation of the cutting force obtained by the experiment is significantly smaller than that in the FEM simulation, which is attributable to the limited sampling frequency of the force dynamometer used in the experiment, resulting in less data points collected compared to the FEM simulation. The average cutting force is obtained by the statistical analysis of averaging the individual values of cutting force in the cutting distance ranging from 0 to 0.3 mm. Specifically, the average cutting force in the FEM simulation is 1.051 N/mm, which deviates from the experimental value of 1.126 N/mm by 6.66%.

**Figure 5:**
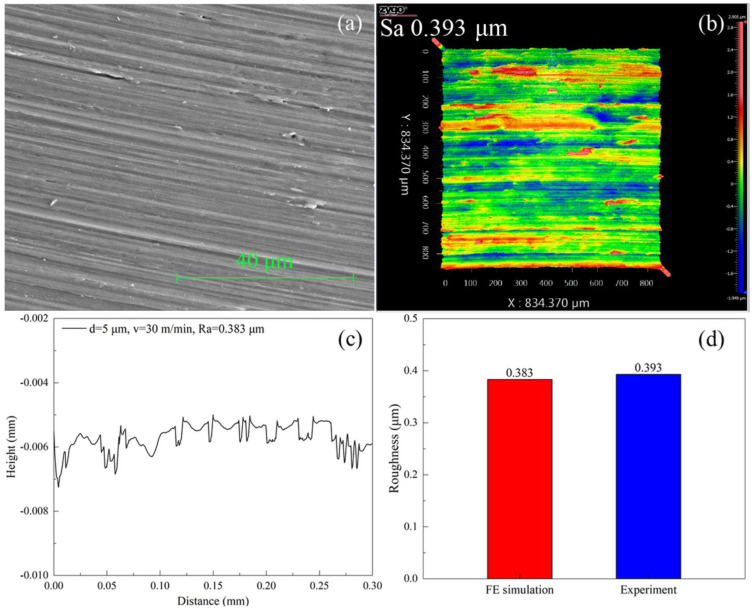
(**a**) SEM image and (**b**) white light interferometer image of machined surface morphology of pure titanium. (**c**) Profile of machined surface by FEM simulation; (**d**) Machined surface roughness in experiment and in FEM simulation.

Figure 5a further presents SEM image of machined surface morphology in the experiment, which shows that the machined surface appears a relatively flat morphology accompanied with shallow tool marks. Figure 5b further shows the machined surface morphology measured with a white light interferometer, indicating a similar shallow tool mark pattern to that in Figure 5a, with a measured surface roughness of 0.393 μm. Figure 5c shows the machined surface profile obtained by FEM simulation, which also demonstrates the presence of periodic fluctuations on the machined surface that are caused by the tool marks. The roughness obtained by FEM simulation is 0.383 μm, which is quantitatively consistent with the experiment of 0.393 μm, as shown in Figure 5d.

### 3.2. Effect of Cutting Depth on Pure Titanium Cutting

**Figure 6:**
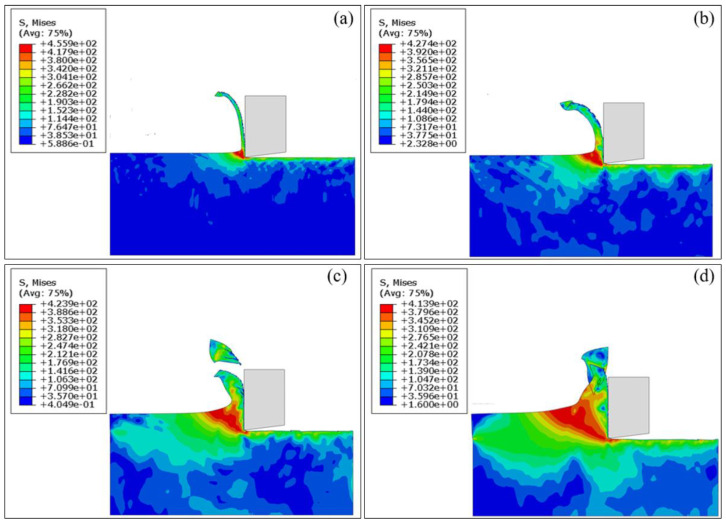
FEM simulation results of cutting configurations of pure titanium at the fixed cutting speed of 10 m/min and different cutting depths: (**a**) 5 μm; (**b**) 10 μm; (**c**) 20 μm; (**d**) 30 μm.

**Figure 7:**
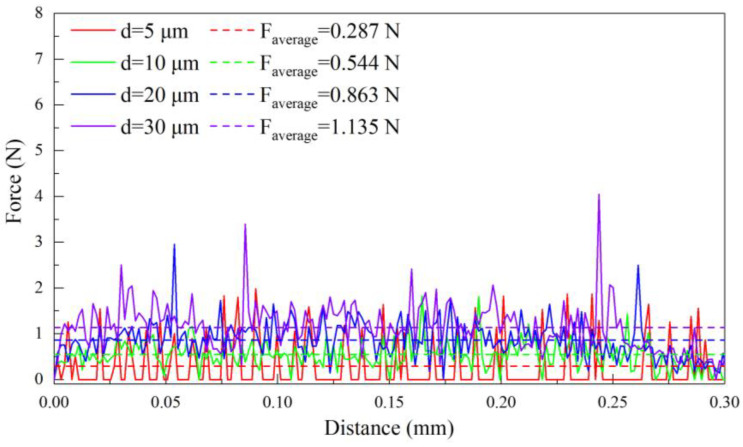
Variation of cutting force over cutting distance in FEM simulation of pure titanium cutting at different cutting depths with the fixed cutting speed of 10 m/min.

Figure 6 presents the cutting configurations of workpiece-tool interaction at cutting depths of 5 μm, 10 μm, 20 μm, and 30 μm, and the cutting speed is fixed as 10 m/min. Figure 7 further plots the variations of cutting force over the cutting distance for each cutting depth. It can be seen in Figure 6a that the amount of workpiece material removed is the smallest at a cutting depth of 5 μm, resulting in the minimum average cutting force of 0.287 N, as shown in Figure 7 (*d* = 5 μm). Furthermore, the stress concentration occurs in the workpiece-tool interface, indicating that the workpiece undergoes obvious plastic deformation with the ribbon chips. The increase in the workpiece-tool interface is accompanied by an increase in the stress concentration area and plastic deformation with a cutting depth to 10 μm, as shown in Figure 6b, accompanied by an 89% increase in the average cutting force of 0.544 N, as shown in Figure 7 (*d* = 10 μm). Figure 6c shows that the stress concentration area of the workpiece increases and shear slip occurs with a cutting depth increasing to 20 μm, resulting in slightly serrated features and fracture in the chips. Correspondingly, the average cutting force is further increased by 59% to 0.863 N, as shown in Figure 7 (*d* = 20 μm). With a further increase in cutting depth to 30 μm, the stress concentration area of the workpiece further increases, as seen in Figure 6d, which results in obvious serrated chips and fracture chips, accompanied by the maximum average cutting force of 1.135 N, in Figure 7 (*d* = 30 μm).

Additionally, Figure 7 (*d* = 5 μm and 10 μm) indicates that the cutting force stability and uniform distribution are observed, which is attributed to the small cutting depth maintaining the stress concentration within the tool-workpiece interface, leading to the formation of ribbon chips and a stable cutting force. However, Figure 7 (*d* = 20 μm and 30 μm) illustrates that the cutting force displays a cyclical behavior of initially increasing and subsequently decreasing at the large cutting depth, which is attributed to the enhanced plastic deformation of the workpiece, peaking when localized shear slippage occurs within the material. As shear slippage progresses, the cutting force exhibits a gradual reduction. The cutting force reaches the minimum when a serrated segment is fully established or a fracture event occurs. This pattern of cutting force variation recurs with the emergence of subsequent serrated segments or fracture events. Moreover, as compared to *d* = 20 μm, *d* = 30 μm is accompanied by a larger cutting depth, leading to denser serrated features of the chips and a smaller variation cycle of cutting force.

**Figure 8:**
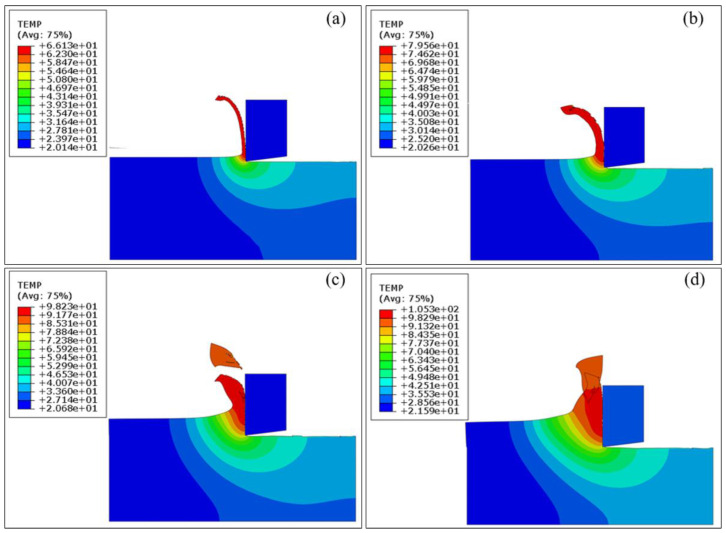
FEM simulation results of cutting temperature of pure titanium at the fixed cutting speed of 10 m/min and different cutting depths: (**a**) 5 μm; (**b**) 10 μm; (**c**) 20 μm; (**d**) 30 μm.

Figure 8 illustrates the distribution of cutting temperature within the workpiece at cutting depths of 5 μm, 10 μm, 20 μm, and 30 μm, and the cutting speed is fixed as 10 m/min. At different cutting depths, the cutting temperature distribution characteristics are generally consistent: the cutting temperature is highest in the workpiece-tool interface and decreases continuously from the peak temperature at the tool tip to the outside. Figure 8a shows that the peak cutting temperature within the cutting area reaches 66.13 °C at the cutting depth of 5 μm. Figure 8b,c show that the peak cutting temperature in the cutting area increases to 79.56 °C at a 10 μm cutting depth with an increase of 20%, and further to 98.23 °C at a 20 μm cutting depth with an increase of 23%. Figure 8d shows that as the cutting depth is increased to 30 μm, the peak cutting temperature in the cutting area reaches a maximum value of 105.31 °C, which is attributed to the resistance that needs to be overcome for the material in the main shear zone to undergo plastic deformation as it also increases with the cutting depth. Simultaneously, the shape of the chips reveals that the tool-chip interface increases with the cutting depth, making it challenging for the chips to be extruded from the front cutting edge. At this time, the heat generation within the cutting system inevitably increases. Furthermore, when the cutting depth increases, the corresponding heat dissipation area increases. However, due to the fast cutting speed and short time in the FEM simulation process, and the fact that this process does not consider the forced heat exchange from the outside (such as cutting fluid cooling, etc.), a comprehensive analysis of thermal generation and thermal dissipation shows that the cutting temperature exhibits a direct proportionality to the cutting depth.

**Figure 9:**
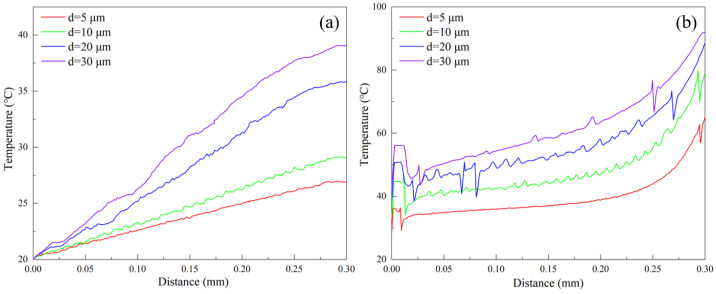
Variation of cutting temperature at (**a**) tool tip and (**b**) workpiece surface over cutting distance in FEM simulations of pure titanium cutting at different cutting depths with the fixed cutting speed of 10 m/min.

Figure 9 further plots variations of cutting temperature for tool tip and workpiece surface over the cutting distance in the FEM simulations at cutting depths of 5 μm, 10 μm, 20 μm, and 30 μm. Figure 9a indicates that the tool tip temperature over the cutting distance increases linearly for each cutting depth, along with the changing rate which gradually increases with the cutting depth. Figure 9b shows that the variation of workpiece surface temperature over the cutting distance is generally consistent with the different cutting depths. The local increase in surface temperature is primarily due to the direct workpiece-tool interaction during the initial cutting stage. Presently, the workpiece-tool contact enters into a steady-state cutting process, resulting in a gradual exponential increase in the workpiece surface temperature, which is the maximum at the tail end of the workpiece. However, Figure 9b (d = 20 μm and 30 μm) illustrates that as the cutting process progresses, the workpiece surface temperature fluctuates irregularly and violently, which is mainly caused by the fracture chips. Furthermore, the workpiece surface temperature at the same cutting distance increases with the cutting depth.

**Figure 10:**
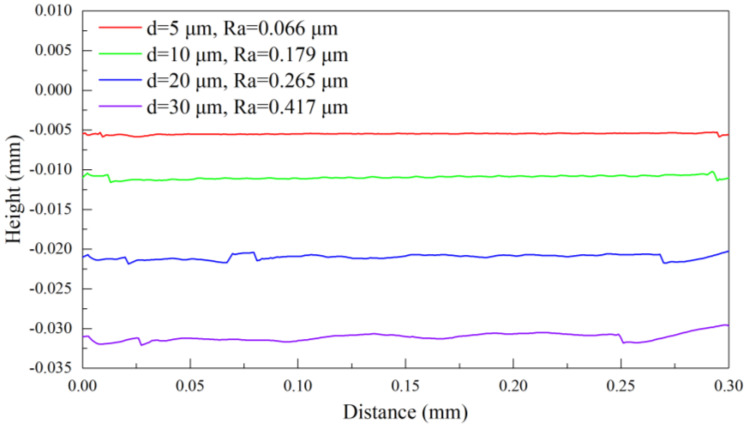
FEM simulation of machined surface profile at different cutting depths with the fixed cutting speed of 10 m/min.

Figure 10 plots the machined surface profile at cutting depths of 5 μm, 10 μm, 20 μm, and 30 μm obtained by FEM simulations of pure titanium cutting. The cutting depth of 5 μm results in a smooth profile with a roughness of 0.066 μm. As the cutting depth is increased to 10 μm, the profile appears comparatively flat and roughness increases to 0.179 μm with an increase of 171%. The plastic deformation becomes more intensified with a cutting depth of 20 μm, which results in the appearance of local protrusions on the profile, accompanied by a roughness of 0.265 μm, reflecting an increase of 48%. At the cutting depth of 30 μm, the workpiece undergoes severe plastic deformation, resulting in irregular undulations on the profile and a maximum roughness of 0.417 μm. Thus, at a fixed cutting speed of 10 m/min, an optimized cutting depth of 5 μm achieves a pure titanium surface with superior quality, characterized by a roughness of 0.066 μm.

### 3.3. Effect of Cutting Speed on Pure Titanium Cutting

**Figure 11:**
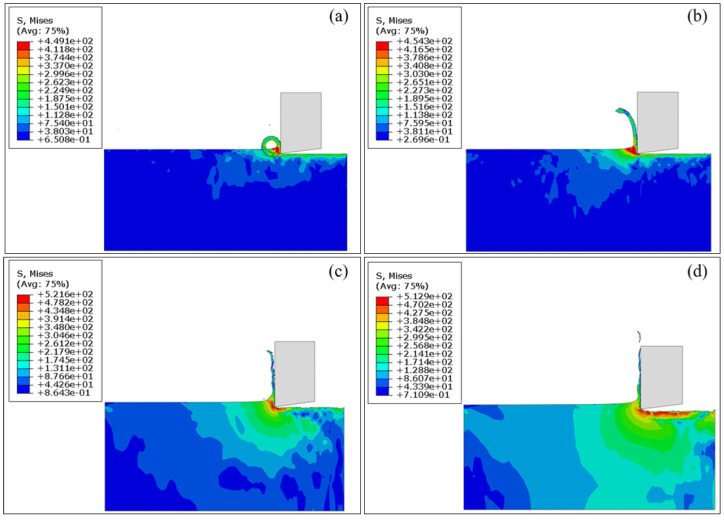
FEM simulation results of cutting configurations of pure titanium at the fixed cutting depth of 5 μm and different cutting speeds: (**a**) 1 m/min; (**b**) 10 m/min; (**c**) 30 m/min; (**d**) 50 m/min.

**Figure 12:**
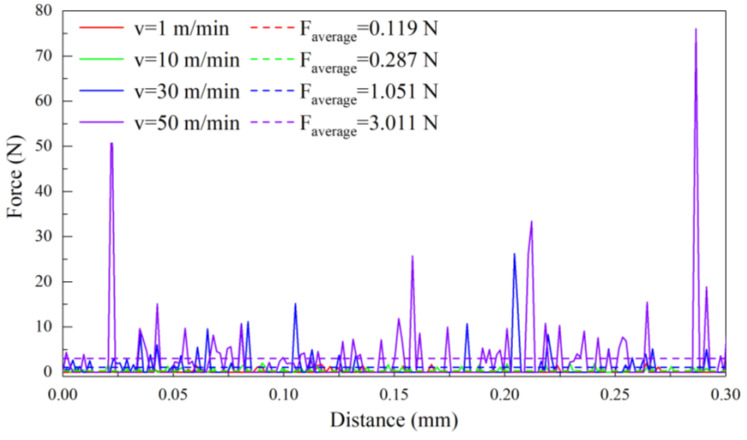
Variation of cutting force over cutting distance in FEM simulations of pure titanium at different cutting speeds with the fixed cutting depth of 5 μm.

To further delve into the effect of cutting speed on the machining performance of pure titanium, Figure 11 shows the simulated cutting configurations of the workpiece-tool interaction at different cutting speeds of 1 m/min, 10 m/min, 30 m/min, and 50 m/min. The cutting depth of 5 μm is fixed with each cutting speed, as seen in Section 3.2. Figure 12 further plots the variation of cutting force over the cutting distance for each cutting speeds. Figure 11a shows that the minimum cutting speed is 1 m/min, resulting in the minimum average cutting force of 0.119 N, as shown in Figure 12 (v = 1 m/min). Furthermore, stress concentration occurs in the workpiece-tool interface, indicating the plastic deformation, accompanied with the spiral ribbon chips. Figure 11b shows that obvious plastic deformation is accompanied by the formation of long strips of ribbon chips as the cutting speed increases to 10 m/min, resulting in an increased average cutting force of 0.287 N in Figure 12 (v = 10 m/min), representing a 141% increase. Figure 11c shows that the stress concentration area shifts from the shear band to the machined surface and the irregular ribbon chips are formed with a cutting speed increasing to 30 m/min, which is attributed to the severe plastic deformation in the shear band, as well as the extrusion effect between the chips and the tool. Correspondingly, the average cutting force is further increased by 266% to 1.051 N, as shown in Figure 12 (v = 30 m/min). The stress concentration area further shifts from the shear band to the machined surface with a cutting speed further increasing to 50 m/min, resulting in the fracture of the irregular ribbon chips, as shown in Figure 11d, accompanied by the maximum average cutting force of 3.011 N, as shown in Figure 12 (v = 50 m/min).

In addition, Figure 12 (v = 1 m/min and 10 m/min) shows that the cutting force appears small and stable, which is attributed to the small cutting speed which causes the stress concentration area to always concentrate in the workpiece-tool interface accompanied with the formation of ribbon chips. However, it can be seen in Figure 12 (v = 30 m/min and 50 m/min) that the cutting force fluctuates strongly in local areas. The cutting force gradually increases with the intensified plastic deformation in the shear band, which is attributed to the higher cutting speed. Subsequently, the peak cutting force appears when internal shear slip is most concentrated within the workpiece accompanied with the accumulated chips on the rake face. Finally, with the continued progression of shear slip and chip fracturing, the cutting force decreases to a minimum value.

**Figure 13:**
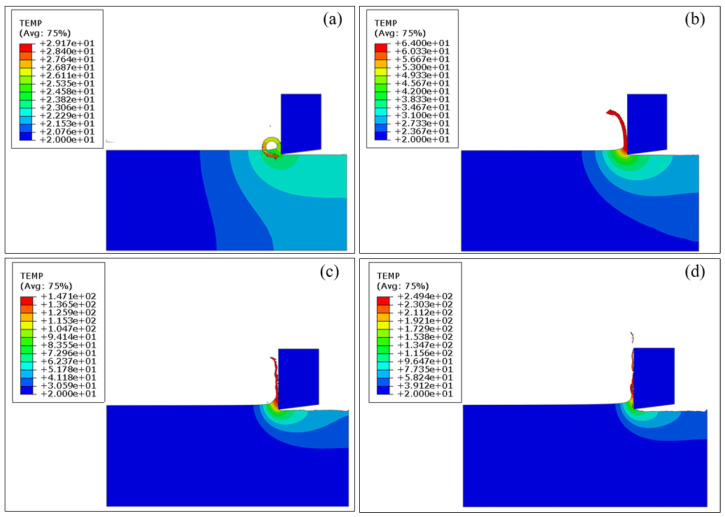
FEM simulation results of cutting temperature of pure titanium at the fixed cutting depth of 5 μm and different cutting speeds: (**a**) 1 m/min; (**b**) 10 m/min; (**c**) 30 m/min; (**d**) 50 m/min.

Figure 13 illustrates the distribution of cutting temperature within the workpiece at different cutting speeds of 1 m/min, 10 m/min, 30 m/min, and 50 m/min. The cutting speed exerts a pronounced impact on the cutting temperature, that is, the temperature state of the entire cutting system increases with the cutting speed. Figure 13a shows that the peak cutting temperature within the cutting area reaches 25.35 °C at the cutting speed of 1 m/min. Furthermore, the temperature of the interface between the spiral ribbon chips and the unmachined workpiece surface increases to 29.17 °C, which is caused by the friction between them. Figure 13b,c illustrates that the peak cutting temperature in the cutting area increases to 64.00 °C at 10 m/min with an increase of 152%, and further to 147.13 °C at 30 m/min with an increase of 130%. Figure 13d illustrates that the peak cutting temperature in the cutting area reaches a maximum value of 172.94 °C at 50 m/min. Furthermore, the high cutting speed causes the chips to accumulate on the rake face, resulting in the temperature of the interface between the chip and the rake face increasing to 249.41 °C. Tool-chip friction intensifies with high cutting speeds, leading to an increase in heat generated within the second deformation zone. Due to the short cutting time and high speed, the heat generated remains in the cutting system due to limited time to dissipate with the external medium, resulting in the increase of the temperature.

**Figure 14:**
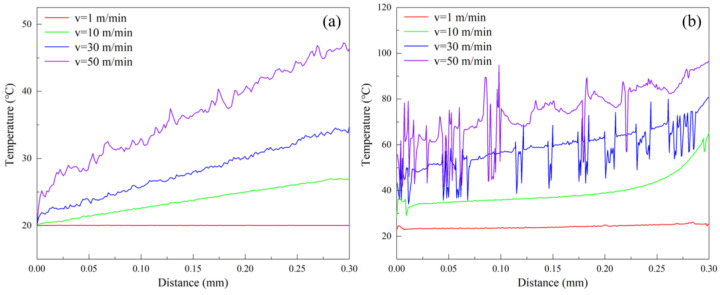
Variation of cutting temperature at (**a**) tool tip and (**b**) workpiece surface over cutting distance in FEM simulations of pure titanium cutting at different cutting speeds with the fixed cutting depth of 5 μm.

Figure 14 further plots variations of cutting temperature of tool tip and workpiece surface over the cutting distance in the FEM simulation at different cutting speeds of 1 m/min, 10 m/min, 30 m/min, and 50 m/min. Figure 14a (v = 1 m/min), b (v = 1 m/min) indicates that the cutting temperature of the tool tip and the workpiece surface remains relatively stable at 20 °C and 23 °C, respectively, at the cutting speed of 1 m/min, which is primarily caused by the smaller cutting force and longer cutting time associated with the lower cutting speed. Additionally, Figure 14a (v = 10 m/min, 30 m/min and 50 m/min) indicates that the tool tip temperature over the cutting distance increases linearly for each cutting speed, and the changing rate of which gradually increases with the cutting speed. Figure 14b (v = 10 m/min, 30 m/min and 50 m/min) shows that the workpiece surface temperature increases locally at the initial stage, which is mainly caused by the direct workpiece–tool interaction in the initial cutting stage. The workpiece surface temperature gradually increases and reaches the maximum at the end of the cutting stage as the cutting process progresses. In addition, Figure 14b (v = 30 m/min and 50 m/min) shows that the workpiece surface temperature increases over the cutting distance, accompanied by irregular and violent fluctuations, which is mainly caused by the extrusion effect between chips and tools and the chip fracturing. Furthermore, increasing the cutting speed causes the workpiece surface temperature to increase at a constant cutting distance.

**Figure 15:**
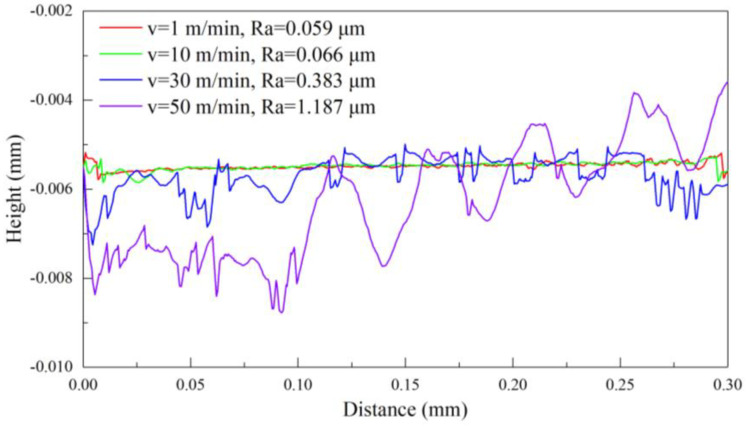
FEM simulation of a machined surface profile of pure titanium at different cutting speeds with the fixed cutting depth of 5 μm.

Figure 15 plots the machined surface profile of pure titanium at different cutting speeds of 1 m/min, 10 m/min, 30 m/min, and 50 m/min in FEM simulations. The cutting peed of 1 m/min results in a smooth profile with a roughness of 0.059 μm. The profile appears comparatively smooth and the roughness increases by 12% to 0.066 μm with the cutting speed of 10 m/min. A smaller cutting speed results in smaller and more stable cutting forces, which in turn allows the tool to cut the workpiece continuously, leading to the ribbon chips and the smaller roughness. The plastic deformation becomes more intensified with irregular ribbon chips forming with a further increase in the cutting speed to 30 m/min, resulting in the appearance of irregular protrusions on the profile with a roughness of 0.383 μm, which represents an increase of 480%. The workpiece undergoes severe plastic deformation at the cutting speed to 50 m/min, leading to the chip fracturing and resulting in severe irregular undulations on the profile, with a maximum roughness of 1.187 μm. Therefore, at a fixed cutting depth of 5 μm, the cutting temperature of the tool tip and workpiece surface reaches the lowest point, and the pure titanium reaches the highest surface quality with a roughness of 0.059 μm through the optimization of the cutting speed at 1 m/min.

### 3.4. Cutting-Based Fabrication of Grid Microtexture on Pure Titanium

As reported in Section 3.2 and Section 3.3, the reduction of the depth and speed in the cutting reduce the surface roughness and the machined surface deformation and also reduce the heat accumulation during the pure titanium cutting. The effect of above depths and speeds in the cutting on the geometry of grid microtextures on pure titanium is further evaluated.

**Figure 16:**
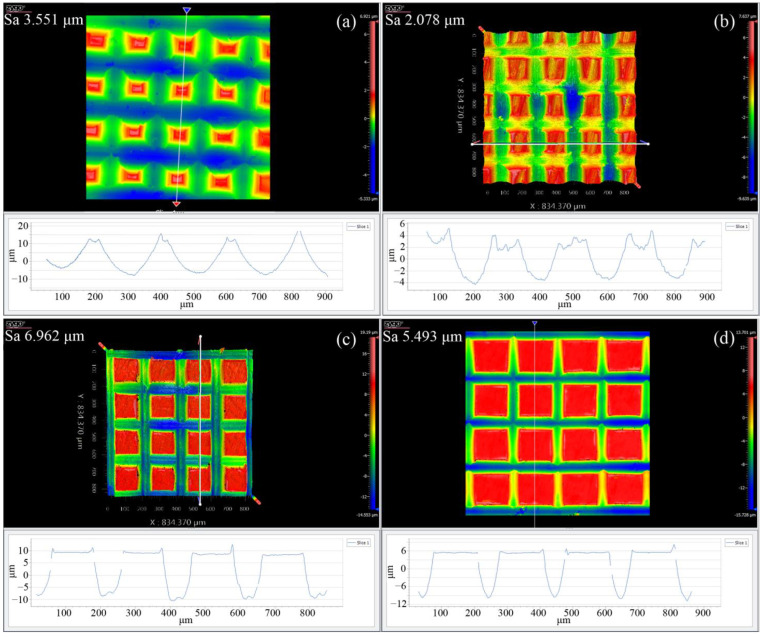
Geometry of grid microtexture fabricated on pure titanium characterized by white light interferometer under different cutting parameters: (**a**) rt = 1 mm, d = 10 μm, v = 1 m/min; (**b**) rt = 50 μm, d = 10 μm, v = 1 m/min; (**c**) rt = 50 μm, d = 10 μm, v = 1 mm/min; (**d**) rt = 50 μm, d = 5 μm, v = 1 mm/min.

Figure 16 presents the geometry of the grid microtexture on pure titanium with a grid depth of 10 μm characterized by white light interferometry under different cutting parameters. Figure 16a illustrates that for the tool tip nose radius at a cutting depth and cutting speed of 1 mm, 10 μm and 1 m/min, respectively, results in a square grid area that is small and irregular, mainly due to the larger grid microtexture width and severe deformation of the grid microtexture edge caused by the larger tool nose radius. Figure 16b indicates that atother fixed cutting parameters, the reduction of the tool nose radius to 50 μm leads to an increase in the square grid area with the irregular shape and the formation of rough defects at the bottom of the grid microtexture, which is attributed to a decrease in the grid microtexture width caused by the reduction of the tool nose radius. However, the high cutting speed leads to defects formed at the bottom of the grid microtexture and severe deformation at the edge of the grid microtexture. Figure 16c shows that the cutting speed is further reduced to 1 mm/min based on the cutting parameters in Figure 16b, and the square grid shape is relatively regular with local irregular deformation at the edge, accompanied by a bulge formed at the bottom of the grid microtexture. The reduction of cutting speed alleviates the deformation of the grid microtexture edge, resulting in a more regular shape of the square grid. However, the larger cutting depth causes the bottom of the grid microtexture to deform seriously, resulting in a bulge formed at the bottom of the grid microtexture by elastic recovery. Figure 16d shows that for the tool tip nose radius, at a cutting depth and cutting speed of 50 μm, 5 μm and 1 m/min, respectively, the grid microtexture depth of 10 μm is achieved through two machining processes, featuring a regular square shape without obvious edge deformation and a flat, smooth bottom with no significant defects.

**Figure 17:**
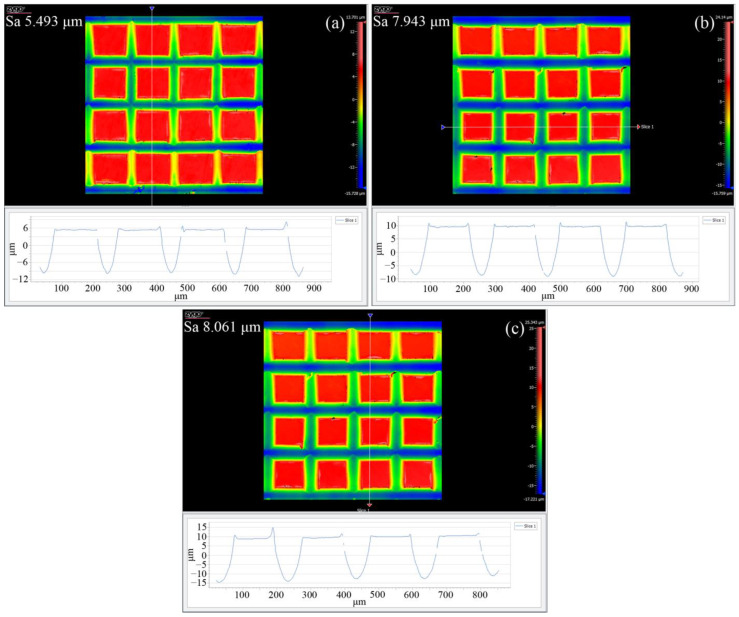
White light interferometer images of grid microtexture fabricated on pure titanium by optimized cutting parameters. The grid depth is: (**a**) 10 μm; (**b**) 20 μm; (**c**) 30 μm.

Figure 17 illustrates the topography of grid microtextures measured with a white light interferometer with different grid depths of 10 μm, 20 μm, and 30 μm and an optimized cutting depth and cutting speed of 5 μm and 1 mm/min, respectively, which demonstrates that for each grid depth, the fabricated grid microtexture has high shape accuracy, superior linearity, and consistent depth, width, and pitch distributions. Specifically, the average depths of microtextures at different grid depths are approximately 13.6 μm, 18.3 μm, and 24.1 μm, with deviations of 26%, 8%, and 19% from the designed values of 10 μm, 20 μm, and 30 μm, respectively, which is mainly caused by the flatness of the workpiece and the elastic recovery of pure titanium after the cutting. Furthermore, the average pitches of the microtexture at different grid depths are approximately 198.8 μm, 199.3 μm, and 200.2 μm, with deviations of 0.6%, 0.3%, and 0.1% from the designed value of 200 μm. The formed geometry of the grid microtexture has a small deviation from the designed geometry, indicating that the high machining accuracy in the pure titanium cutting can be achieved by the optimized cutting parameters.

### 3.5. Surface Wettability of Textured Pure Titanium Surface

**Figure 18:**
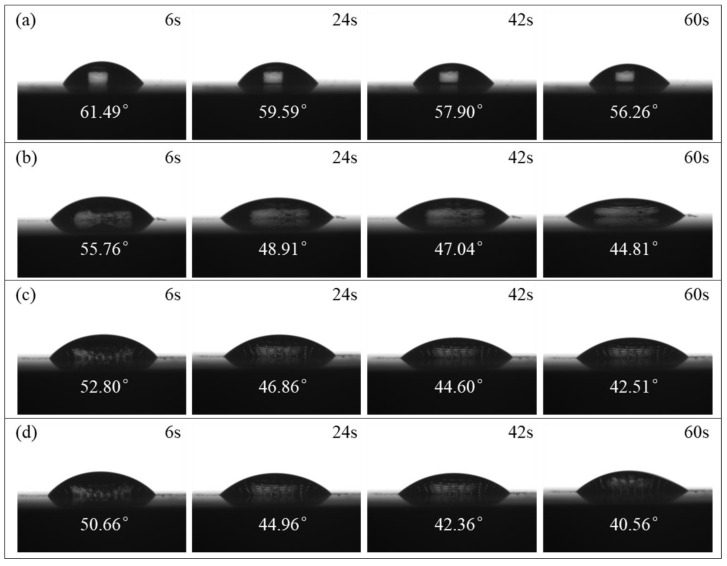
Evolutions of DD water profile over time on pure titanium surface: (**a**) non-microtextured surface; microtextured surface with the grid depth of (**b**)10 μm, (**c**) 20 μm, and (**d**) 30 μm.

Figure 18 experimentally shows the evolution of the DD water profile over time on the non-microtextured pure titanium surface and the microtextured pure titanium surface with different grid depths of 10 μm, 20 μm, and 30 μm, indicating that the DD water contact angle decreases over time on all surfaces, attributed to the reduction in surface tension over time. Furthermore, compared with non-microtextured pure titanium surface, the variation of the coverage area and the height of DD water on the microtextured pure titanium surface changes more significantly with time. DD water on microtextured pure titanium surface has a larger coverage area and a smaller height than that on non-microtextured pure titanium surfaces over the same time. In addition, for the microtextured pure titanium surface with different grid depths, the height of the DD water further decreases and the occupied area of the DD water increases with the grid depth. Thus, with the increase of the grid depth, the DD water contact angle on the microtextured pure titanium surface gradually decreases, all of which are significantly smaller than that on the non-microtextured pure titanium surface.

**Figure 19:**
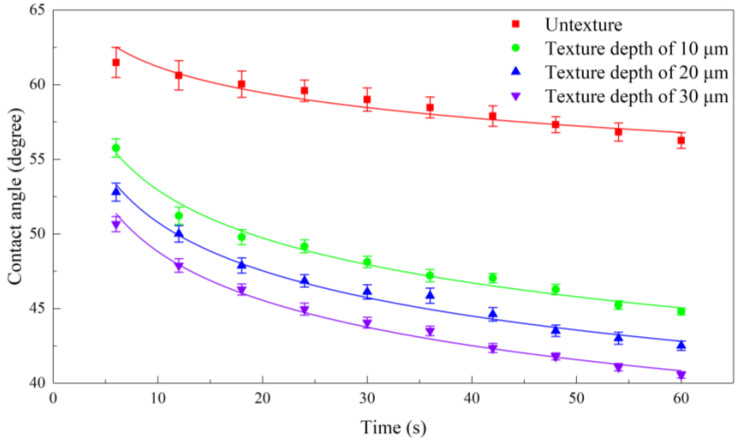
Variations of DD water contact angle over time on non-microtextured and microtextured pure titanium surface with different grid depths of 10 μm, 20 μm and 30 μm.

Figure 19 plots the variation of DD water contact angle over time on the non-microtextured pure titanium surface and the microtextured pure titanium surface with different grid depths of 10 μm, 20 μm, and 30 μm. Compared to the non-microtextured pure titanium surface, the DD water contact angle significantly decreases over time on the microtextured pure titanium surface, which demonstrates a better wettability of the microtextured pure titanium surface than on the non-microtextured pure titanium surface. Furthermore, for the microtextured pure titanium surface with different grid depths, the contact angle of DD water gradually decreases with the increase of grid depth at the same time, indicating that the wettability of the microtextured pure titanium surface increases with the grid depth. The wettability is closely related to the geometric morphology of the surface microtexture. Based on the theory of Wenzel [40], with the hydrophilicity of pure titanium, the capillary force directs the liquid downward, which facilitates easy entry into the microtextures to form a non-composite wettability model, resulting in an increase in droplet diffusion within the microtextures. Furthermore, Wenzel proposed Equation (7) to describe the contact angle of a rough surface:

(7) $$\cos\theta_{r}=r\cos\theta_{s},$$

where r is the surface roughness, $\theta_{r}$ is the corrected rough surface contact angle, and $\theta_{s}$ is the contact angle of a smooth surface. The pure titanium interface contact follows the Wenzel model, where a hydrophilic surface becomes increasingly hydrophilic as the roughness increases. Thus, the wettability of hydrophilic metals is improved by the presence of the grid microtexture, and is further enhanced with the increase of the grid depth.

In order to evaluate the diffusion dynamics of DD water on non-microtextured pure titanium surfaces and microtextured pure titanium surfaces with different grid depths, the contact angle-time curves are fitted by Equation (8) [41]:

(8) $$y\left(x\right)=kx^{n}$$

where *y* is the contact angle, x is the time, and *k* and n represent the empirical coefficients of the initial contact angle and diffusion. It can be seen in Figure 19 that Equation (8) successfully fits all the experimental data, displaying correlation coefficients (R^2^) ranging from 0.9037 to 0.9857. A larger value of the DD water diffusion coefficient n signifies the faster drop diffusion dynamics [42]. The values of n obtained by fitting the experimental data in Figure 19 are listed in Table 4, which demonstrates that the DD water diffusion coefficient for the microtextured pure titanium surface with different grid depths are all higher than that of non-microtextured pure titanium surfaces. Specifically, the diffusion coefficient of 0.09011 for DD water on the microtextured pure titanium surface with a grid depth of 10 μm is approximately 116% higher than that of 0.04164 on the non-microtextured pure titanium surface. With the grid depth increasing to 20 μm, the diffusion coefficient for the DD water is 0.09523, which is increased by 5.68% compared to the grid depth of 10 μm. With the grid depth further increasing to 30 μm, the diffusion coefficient for the DD water is 0.09969, which is increased by 4.68% compared to the grid depth of 20 μm. Thus, the fabricated grid microtexture significantly impacts the liquid diffusion dynamics, which can be attributed to the increase in both total surface area and surface free energy [43]. Moreover, the effect on the liquid diffusion dynamics enhances with the increase of grid depth.

**Figure 20:**
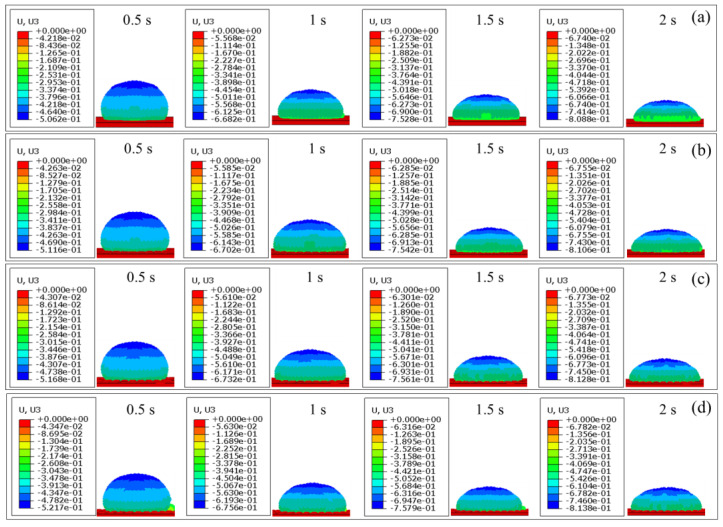
FEM simulation results of displacement evolution of DD water over time on pure titanium surface: (**a**) non-microtextured surface; microtextured surface with the grid depths of (**b**) 10 μm, (**c**) 20 μm and (**d**) 30 μm.

In order to further reveal the wettability behavior of the grid microtexture from a microscopic perspective, Figure 20 illustrates the evolution of displacement of DD water over time on non-microtextured pure titanium surfaces, as well as microtextured pure titanium surfaces with different grid depths of 10 μm, 20 μm, and 30 μm, obtained by FEM simulation. As shown in Figure 20, the displacement of the DD water increases over time, resulting in a decrease in the height and contact angle of DD water on all surfaces over time. When the time is 0.5 s, the height of DD water is 0.9638 mm on the non-microtextured pure titanium surface, and 0.9584 mm, 0.9532 mm, and 0.9483 mm on the microtextured pure titanium surfaces with the increased grid depths. As time increases to 1 s, the height of DD water is 0.8018 mm on the non-microtextured pure titanium surface, and 0.7998 mm, 0.7968 mm, and 0.7944 mm on the microtextured pure titanium surfaces with the increased grid depths. As the time further increases to 1.5 s, the height of DD water is 0.7172 mm on the non-microtextured pure titanium surface, and 0.7158 mm, 0.7139 mm, and 0.7121 mm on the microtextured pure titanium surfaces with the increased grid depths. When the time is 2 s, the height of DD water is 0.6612 mm on the non-microtextured pure titanium surface, and 0.6594 mm, 0.6572 mm, and 0.6562 mm on the microtextured pure titanium surfaces with the increased grid depths. At the same time within 2 s, the height of DD water on microtextured pure titanium surfaces with different grid depths is slightly higher than that on the non-microtextured pure titanium surfaces, and slightly decreases with the increase of the grid depth, which demonstrates that the grid microtexture is conducive to the enhancement of wettability, and the wettability is further enhanced with the increase of grid depth.

**Figure 21:**
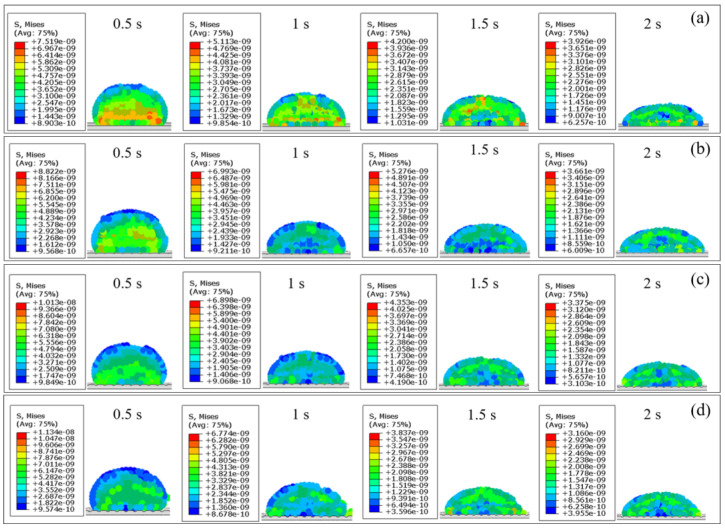
FEM simulation results of stress evolution of DD water over time on pure titanium surfaces: (**a**) non-microtextured surface; microtextured surface with the grid depth of (**b**) 10 μm, (**c**) 20 μm and (**d**) 30 μm.

The wettability is dictated by the cohesive forces of liquid molecules to each other as well as the adhesive forces between liquid molecules and solid molecules. To further study the influences of microtextures with different grid depths on the wettability, Figure 21 presents evolution of stress of DD water over time on non-microtextured pure titanium surfaces and microtextured pure titanium surfaces with different grid depths of 10 μm, 20 μm, and 30 μm obtained by FEM simulation. At the time of 0.5 s, the internal stress of DD water is concentrated between the droplet and the pure titanium surface, and the manifested adhesion enhances the wettability. Maximum adhesion of DD water on the non-microtextured pure titanium surface is 7.519 × 10^−3^ Pa, and the maximum adhesion of DD water on the microtextured pure titanium surface with a grid depth of 10 μm is 8.822 × 10^−3^ Pa, which is increased by 17.33%, resulting in enhanced wettability. As the grid depth increases to 20 μm and 30 μm, the maximum adhesion of DD water on the microtextured pure titanium surface is 1.013 × 10^−2^ Pa and 1.134 × 10^−2^ Pa, respectively, corresponding to an increase of 14.83% and 11.94%, respectively. As the time increases to 1 s, 1.5 s, and 2 s, the internal stress of DD water gradually concentrates inside the droplet, and the manifested cohesive force and the reduced cohesive force jointly lead to enhanced wettability. When the time is 2 s, the maximum cohesive force of DD water on the non-microtextured pure titanium surface is 3.926 × 10^−3^ Pa, and the maximum cohesive force of DD water on the microtextured pure titanium surface with a grid depth of 10 μm is 3.661 × 10^−3^ Pa, which is reduced by 6.749%, further leading to enhanced wettability. As the grid depth increases to 20 μm and 30 μm, the maximum cohesive force of DD water on the microtextured pure titanium surface is 3.375 × 10^−3^ Pa and 3.160 × 10^−3^ Pa, respectively, corresponding to a decrease of 7.812% and 6.371%, respectively. Therefore, according to the internal stress analysis and prediction of DD water on the non-microtextured pure titanium surface and the microtextured pure titanium surface with different grid depths by FEM simulation, the wettability of the microtextured pure titanium surface is obviously better than that of the non-microtextured pure titanium surface, and the wettability of microtextured pure titanium surface increases with the increase of the grid depth.

## 4. Summary

In summary, experiments and FEM simulations were conducted to investigate the formation of accurate grid microtextures on pure titanium by precision cutting, and to assess its impact on surface wettability. During the cutting process of pure titanium, the fluctuations of cutting force around the average value is accompanied with the formation of shallow tool marks on the machined surface. The cutting force, tool tip temperature, machined surface temperature, and machined surface roughness all increase with cutting depth and cutting speed due to the increased deformation of the workpiece, the accumulation of chips, and cutting heat. Under the optimized conditions of a 5 μm cutting depth and a 1 m/min cutting speed, the cutting force, tool tip temperature, and machined surface temperature reaches a minimum value of 0.119 N, 20 °C, and 23 °C, respectively, accompanied with the formation of a smooth surface with a machined surface roughness of 0.059 μm. A high accuracy and a high quality of grid microtexture on pure titanium with grid depths of 10 μm, 20 μm, and 30 μm at a pitch of 200 μm can be achieved by further reducing the tool tip radius to 50 μm and reducing the cutting speed to 1 mm/min. The grid microtexture is effective in enhancing surface wettability, with the enhancement increasing with grid depth due to the increased surface roughness and decreasing cohesive force of DD water to promote droplet diffusion and reduce the contact angle of DD water. Under the optimized grid microstructure of 30 μm of grid depth and 200 μm of grid pitch, the contact angle at 1 min reaches a minimum value of 40.56°, and the diffusion coefficient for the DD water reaches a maximum value 0.09528.

## Figures and Tables

**Table 1:** Chemical constituents of pure titanium.

Element	H	N	C	O	Fe	Ti
Constituent (%)	0.001	0.01	0.011	0.12	0.034	Residual

**Table 2:** Parameters of modified J-C constitutive law of pure titanium.

Notation	*B* (MPa)	*C*	*n*	*α*	*β*	*T_melt_* (°C)	*T_reference_* (°C)	${\dot{\varepsilon }}_{0}$ (s^−1^)
Value	382.39	0.052	0.294	0.021	3.862	1668	150	0.192

**Table 3:** Parameters of J–C damage model of pure titanium.

Notation	*D*_1_	*D*_2_	*D*_3_	*D*_4_	*D*_5_
Value	0.5	3.89	−1.74	0.014	0.95

**Table 4:** Diffusion coefficient n of DD water on pure titanium surface calculated by fitting of experimental data.

Sample	DD Water	
	n	R^2^
Non-microtextured	−0.04164	0.9037
Microtextured with a grid depth of 10 μm	−0.09011	0.9771
Microtextured with a grid depth of 20 μm	−0.09523	0.9809
Microtextured with a grid depth of 30 μm	−0.09969	0.9857

## Data Availability

The data presented in this study are available on request from the corresponding author due to the ongoing project.
